# Case Report: Unusual inferonasal peripheral retinal involvement in ocular toxoplasmosis: a case report with diagnostic and therapeutic implications

**DOI:** 10.3389/fimmu.2026.1884073

**Published:** 2026-08-04

**Authors:** Xiaodong Li, Wei Qin, Hang Long, Yong Chen

**Affiliations:** 1 Department of Ophthalmology, Guizhou University of Traditional Chinese Medicine, Guiyang, China; 2 Department of Ophthalmology, Zhongshan Hospital of Traditional Chinese, Zhongshan, China; 3 Department of Ophthalmology, Tongren Hospital of Traditional Chinese Medicine, Tongren, China; 4 Shaanxi Provincial Cancer Hospital, Affiliated to Xi’an Jiaotong University, Shaanxi, China

**Keywords:** atypical manifestation, case report, ocular toxoplasmosis, panuveitis, peripheral retinal lesion

## Abstract

**Objective:**

To report the clinical characteristics, diagnostic process, and therapeutic outcome of a case of atypical ocular toxoplasmosis involving the nasal peripheral retina, and to provide reference for the differential diagnosis of this disease.

**Methods:**

Clinical data of a 61-year-old female patient with atypical ocular toxoplasmosis in the left eye were retrospectively analyzed, including medical history, ocular examinations, laboratory tests, diagnosis and treatment process, and follow-up results.

**Results:**

The patient presented with “left eye floaters for 3 months”. She was initially diagnosed with left eye panuveitis at an external hospital, and received anti-inflammatory treatment including glucocorticoids. Although symptoms partially relieved, the retinal lesion showed progressive enlargement. After admission, comprehensive examinations revealed a 2–3 papillary diameter (PD) round white lesion in the inferonasal peripheral retina of the left eye, accompanied by flocculent vitreous opacity. Aqueous humor inflammatory factors were significantly elevated: interleukin (IL)-8 121.3 pg/mL, IL-6 371.8 pg/mL, and vascular cell adhesion molecule (VCAM) 15637.8 pg/mL, while nucleic acid tests for herpes viruses in aqueous humor were negative. Combined with the history of cat exposure, positive serum and intraocular fluid Toxoplasma gondii test results, and diagnostic Goldmann-Witmer coefficient, the final diagnosis of atypical ocular toxoplasmosis in the left eye was confirmed. After treatment with pyrimethamine combined with glucocorticoids and adjuvant anti-inflammatory therapy, intraocular inflammation was controlled and the lesion became stable.

**Conclusion:**

Ocular toxoplasmosis can present as atypical peripheral retinal lesions, which is easily confused with acute retinal necrosis and other diseases in clinical practice. Definitive diagnosis can be made by combining pet exposure history, laboratory pathogen testing, and characteristic imaging manifestations. Standard anti-parasitic combined with anti-inflammatory therapy can achieve favorable prognosis.

## 1 Introduction

Toxoplasma gondii is an obligate intracellular protozoan, and Toxoplasma gondii-induced ocular toxoplasmosis is recognized as one of the most common etiologies of infectious posterior uveitis worldwide (1). The prevalence of ocular toxoplasmosis exhibits substantial geographic heterogeneity: the highest incidence rates are reported in Latin America and African countries, while North America, Northern Europe, and Southeast Asia bear a relatively lower disease burden (1, 2). In China, the seroprevalence of T. gondii infection in the general population is less than 10%, and ocular involvement, although relatively rare, carries important clinical significance (3).

The classic clinical presentation of ocular toxoplasmosis is characterized by a focal yellow-white retinochoroiditis lesion in the posterior pole, frequently adjacent to an old chorioretinal scar and accompanied by prominent vitreous inflammation, a pattern classically described as the “headlight in the fog” sign (2, 4). However, accumulating evidence indicates that a considerable proportion of cases present with atypical manifestations, including peripheral retinal lesions, neuroretinitis, punctate outer retinopathy, occlusive retinal vasculitis, and optic neuropathy, which are particularly common in immunocompetent adult patients (4). These atypical phenotypes often lead to diagnostic confusion with other uveitic entities such as acute retinal necrosis, viral retinitis, tuberculosis-associated uveitis, and ocular toxocariasis, resulting in delayed or inappropriate treatment, which may further cause severe and irreversible visual impairment including retinal detachment, macular edema, and optic atrophy. Notably, misdiagnosed cases are often treated with systemic corticosteroids alone without concurrent anti-toxoplasmic therapy, which may exacerbate parasite replication and lead to accelerated progression of retinal lesions (5). Despite growing recognition of these atypical presentations, the clinical characteristics and diagnostic pitfalls of peripheral ocular toxoplasmosis remain underreported in the global population.

Herein, we report a case of atypical ocular toxoplasmosis presenting as an isolated inferonasal peripheral retinochoroidal lesion in an immunocompetent 61-year-old female, who was initially misdiagnosed with idiopathic panuveitis and treated with corticosteroids alone at a local hospital. We elaborate on the stepwise diagnostic workflow, clinical course, and treatment outcome of this case, and discuss the differential diagnostic approach for atypical peripheral ocular toxoplasmosis, aiming to improve clinicians’ awareness of this uncommon manifestation and reduce diagnostic delay. Nevertheless, as a single case report, the present study does not claim novelty in the strict sense. Its primary value lies in describing an uncommon clinical manifestation of ocular toxoplasmosis in the Chinese population, thereby raising clinical awareness and providing a reference for differential diagnosis in similar cases.

## 2 Case presentation

### 2.1 General information

A 61-year-old female patient presented to the ophthalmology department of our hospital in January 2026 with the chief complaint of “floaters in the left eye for 3 months”. Three months prior, the patient developed floaters in her left eye without obvious precipitating factors, accompanied by blurred vision, redness, pain, and distension of the eye, without metamorphopsia, photophobia, or lacrimation. She had no prior history of chronic underlying diseases. Her drug allergy history included sulfonamides and penicillin. Past medical history included vertigo, lumbar and cervical disc herniation, and she had kept a cat for more than 1 year.

### 2.2 Previous treatment course at external hospital

**Figure 1:**
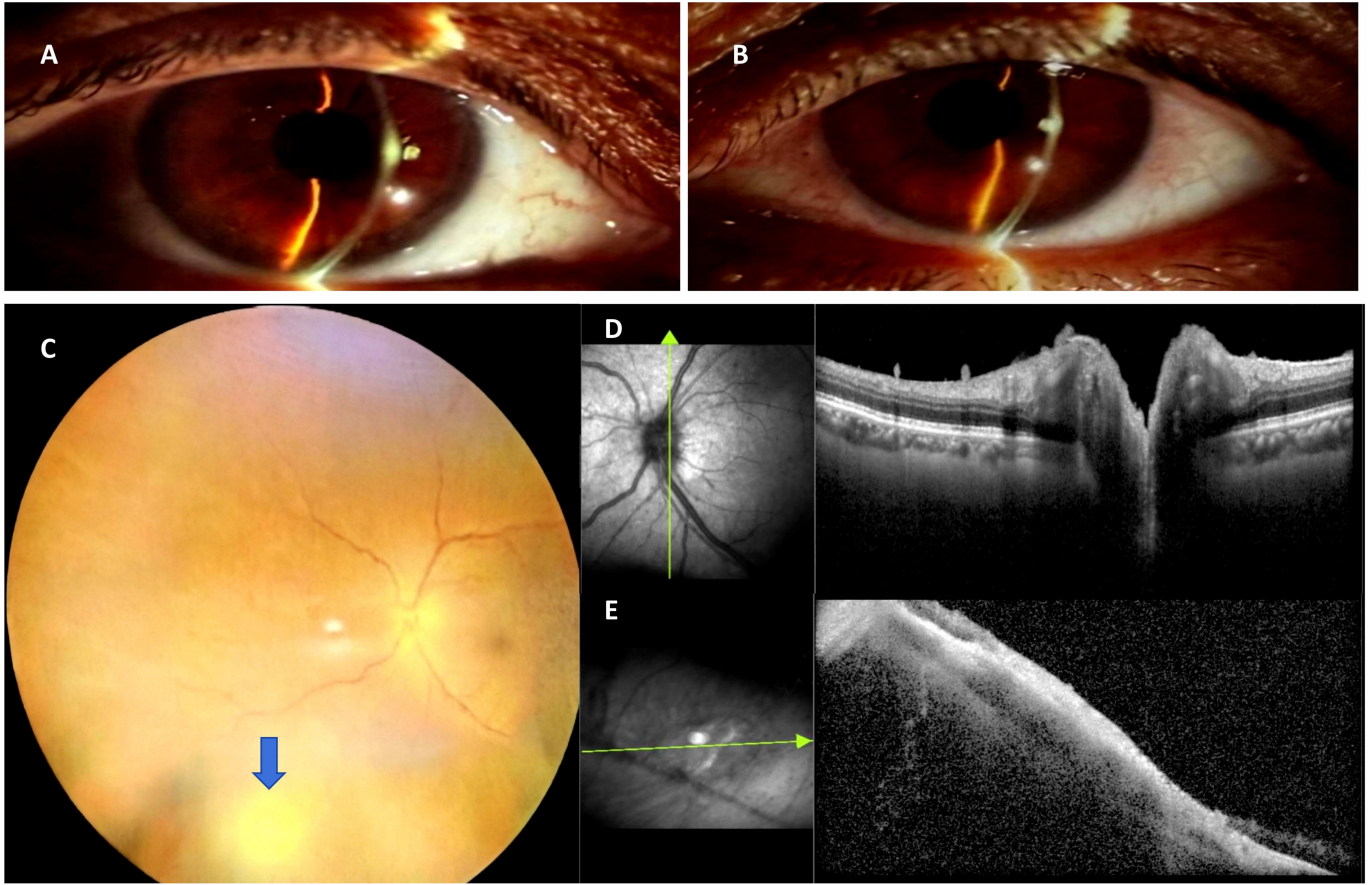
Imaging examinations before treatment. **(A)** Anterior segment photography of the right eye; **(B)** Anterior segment photography of the left eye; **(C)** Color fundus photograph of the left eye (the blue arrow indicates yellowish-white circular lesions); **(D)** OCT image of the left eye optic disc; **(E)** OCT image of the retinal lesion in the left eye.

The patient first visited an ophthalmology clinic at an external hospital in September 2025. At that time, the examination findings were as follows: Visual acuity: best-corrected visual acuity (BCVA) of the right eye was 0.8, BCVA of the left eye was 0.15. Intraocular pressure (IOP) was 12.0 mmHg in both eyes. Anterior segment examination of the left eye showed mixed conjunctival congestion (++), clear cornea, fine keratic precipitates (KP, ++), aqueous flare (++), and aqueous cells (++). The lens presented cortical wedge-shaped opacity, with pigment granules attached to the anterior capsule (**Figures 1A, B**). Posterior segment examination revealed massive flocculent opacities and yellow-white particles in the vitreous cavity. A 1.0 papillary diameter (PD) round yellow-white lesion was observed at the 8:00 o’clock position, 4.0 PD away from the optic disc (**Figure 1C**). Optical coherence tomography (OCT) of the optic disc showed optic disc edema (**Figure 1D**), and OCT of the lesion area demonstrated retinal edema with disorganized structural layers (**Figure 1E**). The external hospital made a preliminary diagnosis of “left eye panuveitis, suspected acute retinal necrosis”. The treatment regimen included: Topical therapy: Prednisolone acetate eye drops, tobramycin and dexamethasone eye ointment, combined with mydriatic treatment. Systemic therapy: Intravenous infusion of dexamethasone 10 mg daily for 5 consecutive days, followed by sequential oral prednisone for anti-inflammatory treatment; oral lecithin complex iodine capsules to improve vitreous opacity; oral acyclovir for antiviral therapy.

During the treatment, the patient developed involuntary tremors of both hands, which were considered to be an adverse reaction to systemic corticosteroid therapy (dexamethasone infusion followed by oral prednisone). Although her visual acuity improved slightly, the floaters showed no significant relief, and the peripheral retinal lesion presented a gradual enlargement trend. Therefore, she was referred to our hospital for further diagnosis and treatment.

## 3 Examination on admission to our hospital

### 3.1 Ocular examination (January 2026)

Examination of the left eye showed BCVA of 0.15,uncorrected visual acuity of 0.25, IOP of 14.0 mmHg. The eyelid showed no redness, swelling, or mass; the conjunctiva had no congestion or edema; the cornea was clear; the anterior chamber was of normal axial depth, with peripheral anterior chamber depth >1/3 corneal thickness (CT). Aqueous cell score was 0, aqueous flare score was 0. The iris texture was clear; the pupil was pharmacologically dilated and round in shape. The lens was opaque, with slight posterior capsule opacity. The vitreous presented flocculent opacity (++) with pigment. The optic disc was pale with blurred borders, cup-to-disc ratio (C/D) = 0.3. The foveal reflex of the macula was absent. The retina appeared grossly flat, with membranous materials observed in the nasal and inferior regions, and a 2–3 PD round white lesion in the inferonasal peripheral retina.

### 3.2 Auxiliary examinations

Routine laboratory tests: Complete blood count, liver and kidney function, blood glucose and lipid profile, four screening tests for infectious diseases, C-reactive protein, erythrocyte sedimentation rate, rheumatoid factor, immunoglobulin panel, antinuclear antibody, antineutrophil cytoplasmic antibody, human leukocyte antigen typing, and anti-double-stranded DNA antibody all showed no abnormalities. Purified protein derivative (PPD) test was negative. The patient was classified as immunocompetent based on the following criteria: (1) complete blood count with differential within normal limits (white blood cell count 6.2 × 10^9^/L, lymphocyte count 1.8 × 10^9^/L, neutrophil count 3.9 × 10^9^/L); (2) normal serum immunoglobulin levels (IgG, IgA, IgM); (3) negative HIV serology; (4) no history of immunosuppressive therapy, immunodeficiency disorders, or chronic systemic diseases (e.g., diabetes, malignancy); and (5) absence of clinical signs suggestive of immunocompromise. Detailed laboratory data supporting immunocompetent status are provided in **Supplementary Table 1**.

Aqueous humor analysis: Pathogenic nucleic acid tests for cytomegalovirus (CMV), Epstein-Barr virus (EBV), herpes simplex virus (HSV), and varicella-zoster virus (VZV) were all negative; fungal 1,3-β-D-glucan was negative. A panel of inflammatory cytokines and soluble mediators was tested in the aqueous humor, including interleukin (IL)-6, IL-8, IL-10, tumor necrosis factor-alpha (TNF-α), interferon-gamma (IFN-γ), and vascular cell adhesion molecule (VCAM). Among these, the following were significantly elevated: IL-8 121.3 pg/mL (reference range 0–20 pg/mL), IL-6 371.8 pg/mL (reference range 1–50 pg/mL), and VCAM 15637.8 pg/mL (reference range 200–1000 pg/mL). The remaining tested mediators (IL-10, TNF-α, IFN-γ) were within normal limits or below the detection threshold. Polymerase chain reaction (PCR) testing for Toxoplasma gondii DNA in aqueous humor was positive (5.78 copies/μL). Inflammatory factors were significantly elevated: interleukin (IL)-8 121.3 pg/mL (reference range 0–20 pg/mL), IL-6 371.8 pg/mL (reference range 1–50 pg/mL),vascular cell adhesion molecule (VCAM)15637.8 pg/mL (reference range 200–1000 pg/mL).

Specific pathogenic tests: both the intraocular fluid Toxoplasma gondii IgG (4.78 U/mL) and serum Toxoplasma gondii IgG (69.84 U/mL) are above the normal reference range, indicating abnormal results. Serum total IgG: 12753.02 U/mL, intraocular fluid total IgG: 216.5 U/mL. GWC is calculated as the ratio of the proportion of specific anti-Toxoplasma IgG to total IgG in intraocular fluid to that in serum. Repeat testing of serum and intraocular fluid for Toxoplasma gondii antibodies showed a calculated Goldmann-Witmer coefficient of 4.03 (diagnostic threshold ≥4), meeting the diagnostic criteria for ocular toxoplasmosis. Tests for Toxocara antibodies and Bartonella henselae (causative agent of cat-scratch disease) were negative; T-SPOT.TB test was negative. Key laboratory findings are summarized in **Table 1**.

**Table 1:** Summary of key laboratory findings.

Parameter	Result	Reference range
Aqueous humor IL-8	121.3 pg/mL	0–20 pg/mL
Aqueous humor IL-6	371.8 pg/mL	1–50 pg/mL
Aqueous humor VCAM	15637.8 pg/mL	200–1000 pg/mL
Intraocular fluidToxoplasma gondiiIgG	4.78 U/mL	Normal: < reference
SerumToxoplasma gondiiIgG	69.84 U/mL	Normal: < reference
Serum total IgG	12753.02 U/mL	—
Intraocular fluid total IgG	216.5 U/mL	—
Goldmann-Witmer coefficient	4.03	> 4: diagnostic
CMV, EBV, HSV, VZV nucleic acid	Negative	Negative
Fungal 1,3-β-D-glucan	Negative	Negative
Aqueous humor T. gondii PCR	5.78 U/mL	Normal: < reference
Toxocaraantibody	Negative	Negative
Bartonella henselaeantibody	Negative	Negative
T-SPOT.TB	Negative	Negative

Imaging examinations: Ultrasound biomicroscopy (UBM) showed deep anterior chamber, open anterior chamber angle, anterior chamber exudates, and lens opacity in the left eye. OCT of the left eye lesion area showed hyperreflectivity of the full-thickness retina, disorganized structure, destruction of outer retinal layers, and focal choroidal thickening. Serial fundus photography showed progressive irregular enlargement of the inferonasal peripheral retinal lesion from September to November 2025.

### 3.3 Final diagnosis

Atypical ocular toxoplasmosis of the left eye (active).

## 4 Treatment regimen

After confirmed diagnosis, standardized anti-Toxoplasma combined with anti-inflammatory therapy was administered. Anti-parasitic therapy: Due to the patient’s sulfonamide allergy, oral pyrimethamine (25 mg/tablet, 50 mg once daily), with supplementary folinic acid (calcium folinate, leucovorin) 10 mg orally every other day to mitigate pyrimethamine-induced bone marrow suppression without reversing the antifolate effect against Toxoplasma gondii. Routine monitoring of blood routine and liver and kidney function was performed during treatment. Anti-inflammatory therapy: Oral prednisone acetate 40 mg was administered in the morning, with gradual tapering of 5 mg every 1–2 weeks. Topical anti-inflammatory therapy included prednisolone acetate eye drops 4 times daily, tobramycin and dexamethasone eye ointment once nightly, and pranoprofen eye drops 4 times daily. Tropicamide phenylephrine eye drops were used for mydriasis to prevent posterior synechia of the iris. Adjuvant therapy: Oral potassium chloride sustained-release tablets and calcium carbonate D3 tablets were given to prevent adverse reactions of corticosteroids; rebamipide was administered for gastric mucosal protection; vitamin B complex and cobamamide were used for neurotrophic support.

## 5 Follow-up and outcome

**Figure 2:**
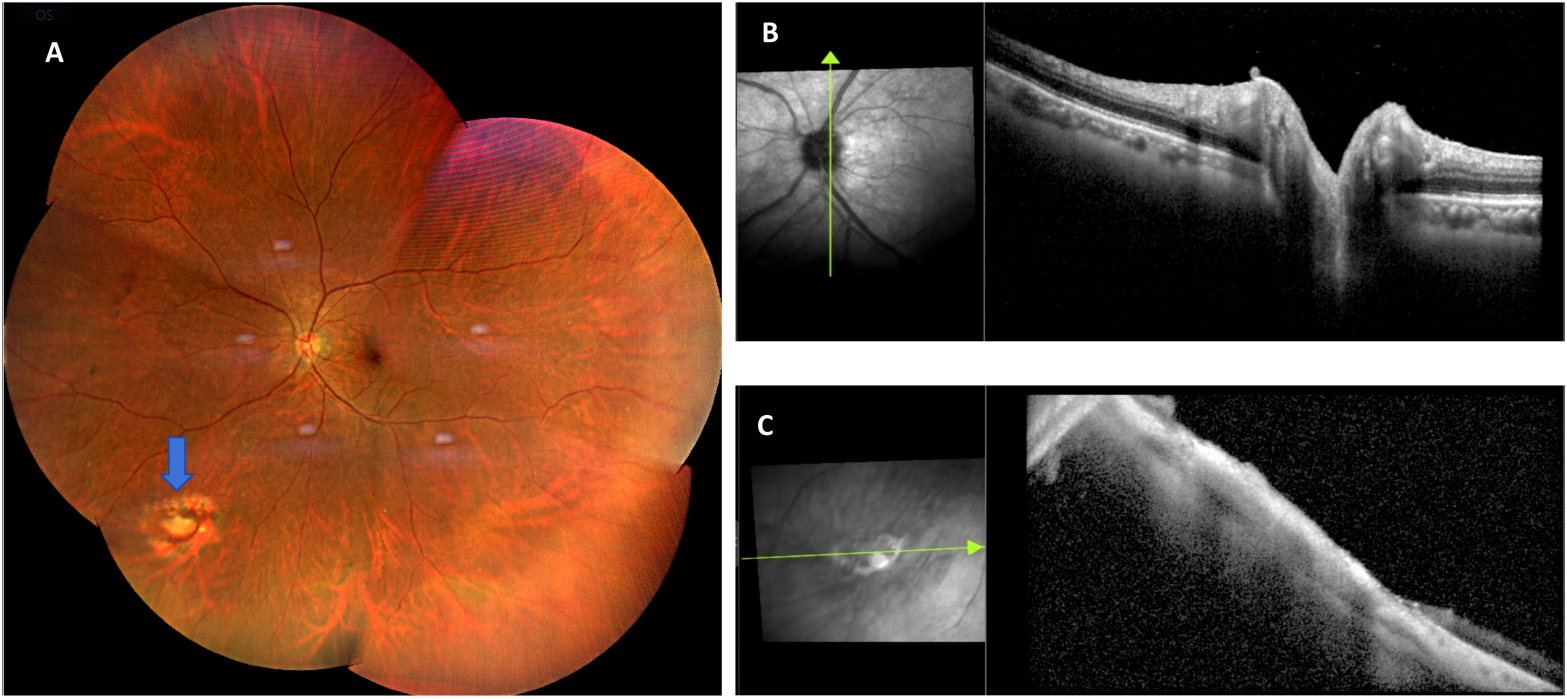
Imaging examinations 4 months after treatment. **(A)** Ultra-wide-angle fundus photography of the left eye (the blue arrow indicates atrophic lesions); **(B)** OCT image of the optic disc in the left eye; **(C)** OCT image of the retinal lesion in the left eye.

Follow-up at 2 months after treatment initiation (March 11, 2026): Left eye visual acuity was 0.5, IOP was 10 mmHg. Slit-lamp examination showed pigmented KP (+) in the cornea, aqueous cells (-), aqueous flare (±). Vitreous opacity was graded as ++, and the retinal lesion remained stable. Follow-up at 4 months after treatment initiation (May 5, 2026): Left eye visual acuity improved to 0.6, IOP was 12 mmHg. Vitreous opacity was reduced to (+). The inferonasal peripheral retinal lesion showed pigmentation and tended to atrophy (**Figure 2A**). Optic disc OCT showed resolution of optic disc edema (**Figure 2B**), and OCT of the lesion area demonstrated retinal atrophy and thinning with disorganized structural layers (**Figure 2C**). The intraocular inflammation was effectively controlled. The patient is currently under regular follow-up, continuing anti-parasitic therapy and low-dose corticosteroid maintenance treatment, with close monitoring of lesion changes. Serial clinical and visual outcomes are summarized in **Table 2**.

**Table 2:** Visual acuity and clinical course over time.

Time point	BCVA (left eye)	IOP (mmHg)	Vitreous opacity	Lesion characteristics
Initial presentation (external hospital, Sep 2025)	0.15	12.0	Massive flocculent opacities	1.0 PD round yellow-white lesion, inferonasal periphery
Admission (Jan 2026)	0.15 (BCVA)/0.25 (UCVA)	14.0	Flocculent opacity (++) with pigment	2–3 PD round white lesion, inferonasal periphery
2-month follow-up (Mar 11, 2026)	0.5	10.0	++	Lesion stable
4-month follow-up (May 5, 2026)	0.6	12.0	+	Lesion pigmented, tending to atrophy

## 6 Discussion

### 6.1 Clinical characteristics and pathogenic mechanisms of ocular toxoplasmosis

Ocular toxoplasmosis, caused by Toxoplasma gondii infection, is a common infectious posterior uveitis with significant geographic heterogeneity in global prevalence. The seropositivity rate in the general Chinese population is less than 10%, making clinical cases relatively rare (1). Classic ocular toxoplasmosis typically presents as a yellow-white retinochoroidal lesion in the posterior pole, adjacent to an old chorioretinal scar, accompanied by prominent vitreous inflammation, which is known as the characteristic “headlight in the fog” sign (2, 4). Current studies (6–9) suggest that the predilection of Toxoplasma gondii lesions for the posterior pole retina, especially the macular region, is related to several mechanisms. First, and most critically, blood containing Toxoplasma gondii flows directly to the end of blood vessels. The posterior pole retina is the initial distribution area of the main branches of the central retinal artery, with high blood flow volume and rapid flow velocity, making it easier for parasites to “colonize”, penetrate the vascular wall, and invade retinal tissue. Second, the tachyzoites of Toxoplasma gondii preferentially invade metabolically active, nutrient-rich tissues. The posterior pole retina, particularly the macular region, contains dense photoreceptors with extremely high metabolic rate and abundant blood supply, providing an ideal “microenvironment” for Toxoplasma gondii replication. Third, intraocular tissues, especially the retina and uvea, are relatively immune-privileged sites. This characteristic also makes latent Toxoplasma gondii cysts difficult to be completely eliminated by the immune system, rendering them more prone to reactivation when immunity declines. As the functional core region, the posterior pole retina may have more prominent immune privilege characteristics. Finally, for congenital toxoplasmosis, retinal development is still incomplete during fetal life, and the vascularization process is ongoing. The posterior pole is the last region of the retina to complete vascularization, which may make it more susceptible to infection and colonization by trans placentally transmitted Toxoplasma gondii during development. However, accumulating evidence (10–13) shows that 15%–20% of immunocompetent patients may present with atypical clinical manifestations, including peripheral retinal lesions, neuroretinitis, punctate outer retinopathy, and other entities. These cases are easily misdiagnosed as other types of uveitis, leading to delayed treatment.

The present case involves an immunocompetent elderly female who presented with “floaters in the left eye for 3 months”. The initial presentation at the external hospital was panuveitis with an isolated yellow-white lesion in the inferonasal peripheral retina, which was inconsistent with the typical characteristics of posterior pole lesions. Combined with the negative aqueous humor viral test results at that time, the patient was misdiagnosed with idiopathic panuveitis and treated with glucocorticoids alone. Although the patient’s inflammatory symptoms partially relieved, progressive enlargement of the retinal lesion and no significant improvement in vitreous opacity were observed during follow-up, suggesting a poor response to conventional anti-inflammatory therapy. After referral to our hospital, detailed medical history collection revealed that the patient had a 1-year history of cat ownership, which highly suggested the possibility of zoonotic infection. Further etiological testing of aqueous humor and serum showed a Goldmann-Witmer coefficient >4, which met the diagnostic criteria for ocular toxoplasmosis. Tests for herpesvirus in aqueous humor, Bartonella henselae, and tuberculosis-related tests were all negative, and the final diagnosis was atypical peripheral ocular toxoplasmosis. The diagnostic process of this case suggests that when encountering patients with unexplained uveitis, especially those with isolated peripheral retinal yellow-white lesions, clinicians should routinely inquire about pet exposure history, include ocular toxoplasmosis in the differential diagnosis spectrum, and avoid the blind use of high-dose glucocorticoid monotherapy or misdiagnosis as viral uveitis with subsequent long-term high-dose antiviral treatment. Of note, the ocular examination on admission revealed a pale optic nerve head and absence of foveal reflex in the left eye. The optic disc pallor is likely attributable to the prior episode of optic disc edema secondary to severe intraocular inflammation observed during the initial presentation at the external hospital, while the absence of foveal reflex may reflect macular involvement secondary to the inflammatory process, even in the absence of a direct macular lesion.

The incidence of atypical peripheral lesions of ocular toxoplasmosis is relatively low, and its anatomical mechanism differs from that of posterior pole lesions. The traditional view holds that Toxoplasma gondii tachyzoites preferentially invade the metabolically active, highly vascularized posterior pole retina. The high metabolic level of the macular region, hemodynamic characteristics of the central retinal artery, and local immune-privileged environment collectively contribute to the susceptibility of the posterior pole (9). However, recent studies have revealed that certain Toxoplasma gondii strains may exhibit higher adhesiveness to peripheral retinal vascular endothelial cells. Meanwhile, the local immune response in immunocompetent hosts may restrict the spread of pathogens to the posterior pole, thus manifesting as isolated peripheral lesions (14, 15). In the present case, the lesion gradually enlarged from an initial size of 1.0 papillary diameter (PD) to 2–3 PD, and was exclusively located in the inferonasal peripheral region without posterior pole involvement, which is consistent with the clinical characteristics of atypical ocular toxoplasmosis. This also indicates that peripheral lesions are similarly progressive, and may extend toward the visual axis without timely intervention, leading to visual function impairment.

### 6.2 Diagnostic evaluation

The diagnosis of atypical ocular toxoplasmosis is primarily based on laboratory examination of patient serum, aqueous humor, and vitreous. The Goldmann-Witmer coefficient, calculated by the ratio of anti-Toxoplasma gondii IgG to total IgG in intraocular fluid and serum detected via enzyme-linked immunosorbent assay (ELISA), is regarded as the laboratory “gold standard” for the clinical diagnosis of ocular toxoplasmosis. A Goldmann-Witmer coefficient below 2 rules out the possibility of ocular toxoplasmosis, a value between 2 and 4 indicates suspected ocular toxoplasmosis, and a value above 4 confirms the diagnosis, with a sensitivity ranging from 39% to 81%. Meanwhile, polymerase chain reaction (PCR) detection of Toxoplasma gondii DNA in intraocular fluid is also used for diagnosis, with a sensitivity of approximately 27%–67% in immunocompetent individuals, which can increase to 75% in immunodeficient patients (1, 2). The combined use of the above two detection methods can improve the laboratory diagnostic rate of ocular toxoplasmosis. According to literature reports (16), Western blot (WB) analysis of serum and paired aqueous humor has similar sensitivity but higher specificity (>95%) compared with the Goldmann-Witmer coefficient. In the present case, the markedly elevated IL-6 and IL-8 levels in the aqueous humor are consistent with the pro-inflammatory cytokine profile typically observed in active ocular toxoplasmosis, while the normal/low levels of other tested mediators may reflect the localized nature of the inflammatory response or the influence of prior corticosteroid therapy. In addition, previous studies have reported that ocular toxoplasmosis patients with an aqueous humor IFN-γ/IL-10 ratio < 1 have a longer disease course and more severe complications (17), suggesting that this ratio may serve as a prognostic predictor. Of note, this parameter was not measured in the present case, as the referenced finding is derived from the cited literature rather than from our patient data.

### 6.3 Differential diagnosis

The differential diagnosis of this case focuses on the following three categories of diseases: Acute retinal necrosis (ARN): This disease is mostly caused by herpesvirus infection, and typically presents as multifocal white necrotic lesions in the peripheral retina that rapidly coalesce and progress toward the posterior pole, accompanied by prominent retinal arteritis and hemorrhage. Nucleic acid testing for herpesvirus in aqueous humor is positive (18). In this case, the lesion was an isolated round lesion without hemorrhage, and herpesvirus testing was negative, which does not support the diagnosis of ARN. Ocular toxocariasis: This condition is common in children, and most patients have a history of cat exposure. Fundus examination shows granulomatous lesions, often accompanied by significant eosinophilia (19). In this case, the serum anti-Toxocara antibody was negative, ruling out this diagnosis. Tuberculous uveitis: Most patients are accompanied by systemic tuberculosis intoxication symptoms, with positive PPD and T-SPOT tests, and multifocal choroidal lesions can be observed on fundus examination (20). All tuberculosis-related tests in this case were negative, ruling out this diagnosis. In addition, infectious diseases such as cat-scratch disease and syphilitic uveitis should be excluded one by one through pathogenic testing.

### 6.4 Treatment and outcome

The core treatment goals for ocular toxoplasmosis are to control inflammation, preserve visual function, and reduce recurrence, with strict adherence to treatment indications. Toxoplasmic retinochoroiditis in immunocompetent individuals is self-limiting, with spontaneous resolution within 4–8 weeks. However, active intervention is required under the following circumstances: lesions located within the retinal vascular arcade, lesions adjacent to the optic disc, or lesions with a diameter > 2 papillary diameters (PD) with extensive inflammation, which are prone to cause vitreous opacity and retinal detachment. Mandatory treatment is indicated for immunocompromised patients (HIV infection, post-organ transplantation) and elderly patients regardless of lesion size (21). The classic treatment regimen has definite efficacy but poor tolerability: the combination of pyrimethamine, sulfadiazine, and glucocorticoids exerts its effect by blocking nucleotide metabolism in trophozoites. However, pyrimethamine may cause bone marrow suppression, and sulfadiazine may induce allergic reactions, requiring close monitoring of blood routine, liver, and renal function in clinical practice. Alternative regimens have comparable efficacy and better safety, and the commonly used alternative treatment regimens in clinical practice are as follows: 1. Clindamycin: 600 mg orally 4 times daily; for severe cases, intravitreal injection of 1 mg clindamycin combined with 0.4 mg dexamethasone can rapidly control intraocular inflammation. 2. Trimethoprim-sulfamethoxazole (TMP-SMX): 2 tablets orally twice daily, which has both therapeutic effects on active infection and preventive effects against recurrence. 3. Azithromycin: 500 mg orally once daily, suitable for patients with sulfonamide allergy. 4. Atovaquone: 750 mg orally twice daily, which has certain activity against Toxoplasma gondii cysts and is preferentially used in immunodeficient patients. 5. Spiramycin: 1 g orally 4 times daily, which is a safe choice for pregnant women and children with congenital infection (22). Meanwhile, after cure, immunocompetent patients can take 1 tablet of TMP-SMX daily for maintenance to reduce the risk of recurrence; immunodeficient patients require long-term monitoring and maintenance treatment.

The present case involved an elderly female patient with severe ocular symptoms who showed no significant relief after initial intervention. Additionally, she had a history of sulfonamide allergy. Therefore, the classic regimen of pyrimethamine combined with sulfadiazine was not used, and pyrimethamine monotherapy combined with glucocorticoids was selected, supplemented by folinic acid (calcium folinate) and gastric mucosal protectants to reduce adverse drug reactions. Folinic acid, rather than folic acid, was used because it selectively rescues host cells from pyrimethamine-induced folate antagonism without providing preformed folate that would circumvent the antiparasitic effect of the antifolate drug on T. gondii proliferation. After 2 months of treatment, the patient’s BCVA improved from 0.15 to 0.5, and after 4 months, it further improved to 0.6. The lesion showed pigmentation and tended to atrophy, with stable disease, confirming the safety and efficacy of this regimen. Previous evidence-based medicine studies have shown that for patients with sulfonamide allergy, regimens such as pyrimethamine monotherapy, clindamycin, and azithromycin have no significant difference in efficacy compared with the classic therapy (23), and the treatment outcome of this case is consistent with literature reports. In addition, on the basis of effective anti-toxoplasmic treatment, this case was treated with topical prednisolone acetate eye drops or oral prednisone to reduce tissue damage caused by inflammatory reactions. Meanwhile, the glucocorticoid dosage was gradually tapered, avoiding the risk of pathogen diffusion that may be caused by the use of glucocorticoids alone, which also conforms to the treatment principles for ocular toxoplasmosis.

## 7 Conclusion

Ocular toxoplasmosis remains an important cause of blindness in many countries, with a significant impact on quality of life. Patients with typical clinical features are more likely to receive timely diagnosis and necessary treatment, leading to improved prognosis. In contrast, atypical ocular toxoplasmosis may present with complex and diverse manifestations, with a wide range of severity, and may even be accompanied by severe vision-threatening complications. Clinicians need to fully understand the disease pathology and recognize that inflammation can easily involve a wide range of intraocular tissues including the vitreous, optic nerve, and macula. The clinical implications of this case are as follows: 1. Atypical ocular toxoplasmosis can manifest as an isolated peripheral retinal lesion without typical posterior pole scarring, which easily leads to misdiagnosis. 2. For patients with unexplained uveitis, it is necessary to routinely collect pet exposure history, and perform serum and intraocular fluid Goldmann-Witmer coefficient testing and pathogenic screening as early as possible to avoid glucocorticoid monotherapy. 3. For patients with sulfonamide allergy, pyrimethamine monotherapy combined with glucocorticoids can achieve favorable therapeutic efficacy. This case enriches the clinical phenotype spectrum of atypical ocular toxoplasmosis in the Chinese population and provides a reference for the differential diagnosis and treatment selection of this disease.

## Data Availability

The raw data supporting the conclusions of this article will be made available by the authors, without undue reservation.
