# Supplementary material for: Case Report: Unusual inferonasal peripheral retinal involvement in ocular toxoplasmosis: a case report with diagnostic and therapeutic implications
Source: Front Immunol. 2026 Aug 4;17:1884073. doi: 10.3389/fimmu.2026.1884073 (PMC13481170; doi:10.3389/fimmu.2026.1884073)
Supplement: Supplementary file 1 [file Table1.docx]

**Supplementary Table S1. Laboratory findings supporting immunocompetent status**

| **Parameter** | **Result** | **Reference range** |
| --- | --- | --- |
| **White blood cell count** | 6.2 × 10⁹/L | 3.5–9.5 × 10⁹/L |
| **Lymphocyte count** | 1.8 × 10⁹/L | 1.1–3.2 × 10⁹/L |
| **Neutrophil count** | 3.9 × 10⁹/L | 1.8–6.3 × 10⁹/L |
| **Hemoglobin** | 128 g/L | 115–150 g/L |
| **Platelet count** | 235 × 10⁹/L | 125–350 × 10⁹/L |
| **Serum IgG** | 12.75 g/L | 7.0–16.0 g/L |
| **Serum IgA** | 2.1 g/L | 0.7–4.0 g/L |
| **Serum IgM** | 1.3 g/L | 0.4–2.3 g/L |
| **HIV serology** | Negative | Negative |
| **C-reactive protein** | 3.2 mg/L | <5.0 mg/L |
| **Erythrocyte sedimentation rate** | 12 mm/h | 0–20 mm/h |
